# Potential implications of a monosynaptic pathway from mossy cells to adult-born granule cells of the dentate gyrus

**DOI:** 10.3389/fnsys.2015.00112

**Published:** 2015-08-19

**Authors:** Helen E. Scharfman, Hannah L. Bernstein

**Affiliations:** ^1^The Nathan Kline Institute for Psychiatric Research, OrangeburgNY, USA; ^2^New York University Langone Medical Center, New YorkNY, USA

**Keywords:** hippocampus, adult neurogenesis, pattern separation, spatial memory, depression, seizure, psychiatry, epilepsy

## Abstract

The dentate gyrus (DG) is important to many aspects of hippocampal function, but there are many aspects of the DG that are incompletely understood. One example is the role of mossy cells (MCs), a major DG cell type that is glutamatergic and innervates the primary output cells of the DG, the granule cells (GCs). MCs innervate the GCs as well as local circuit neurons that make GABAergic synapses on GCs, so the net effect of MCs on GCs – and therefore the output of the DG – is unclear. Here we first review fundamental information about MCs and the current hypotheses for their role in the normal DG and in diseases that involve the DG. Then we review previously published data which suggest that MCs are a source of input to a subset of GCs that are born in adulthood (adult-born GCs). In addition, we discuss the evidence that adult-born GCs may support the normal inhibitory ‘gate’ functions of the DG, where the GCs are a filter or gate for information from the entorhinal cortical input to area CA3. The implications are then discussed in the context of seizures and temporal lobe epilepsy (TLE). In TLE, it has been suggested that the DG inhibitory gate is weak or broken and MC loss leads to insufficient activation of inhibitory neurons, causing hyperexcitability. That idea was called the “dormant basket cell hypothesis.” Recent data suggest that loss of normal adult-born GCs may also cause disinhibition, and seizure susceptibility. Therefore, we propose a reconsideration of the dormant basket cell hypothesis with an intervening adult-born GC between the MC and basket cell and call this hypothesis the “dormant immature granule cell hypothesis.”

## Fundamental Organization of the Dentate Gyrus (DG)

**FIGURE 1:**
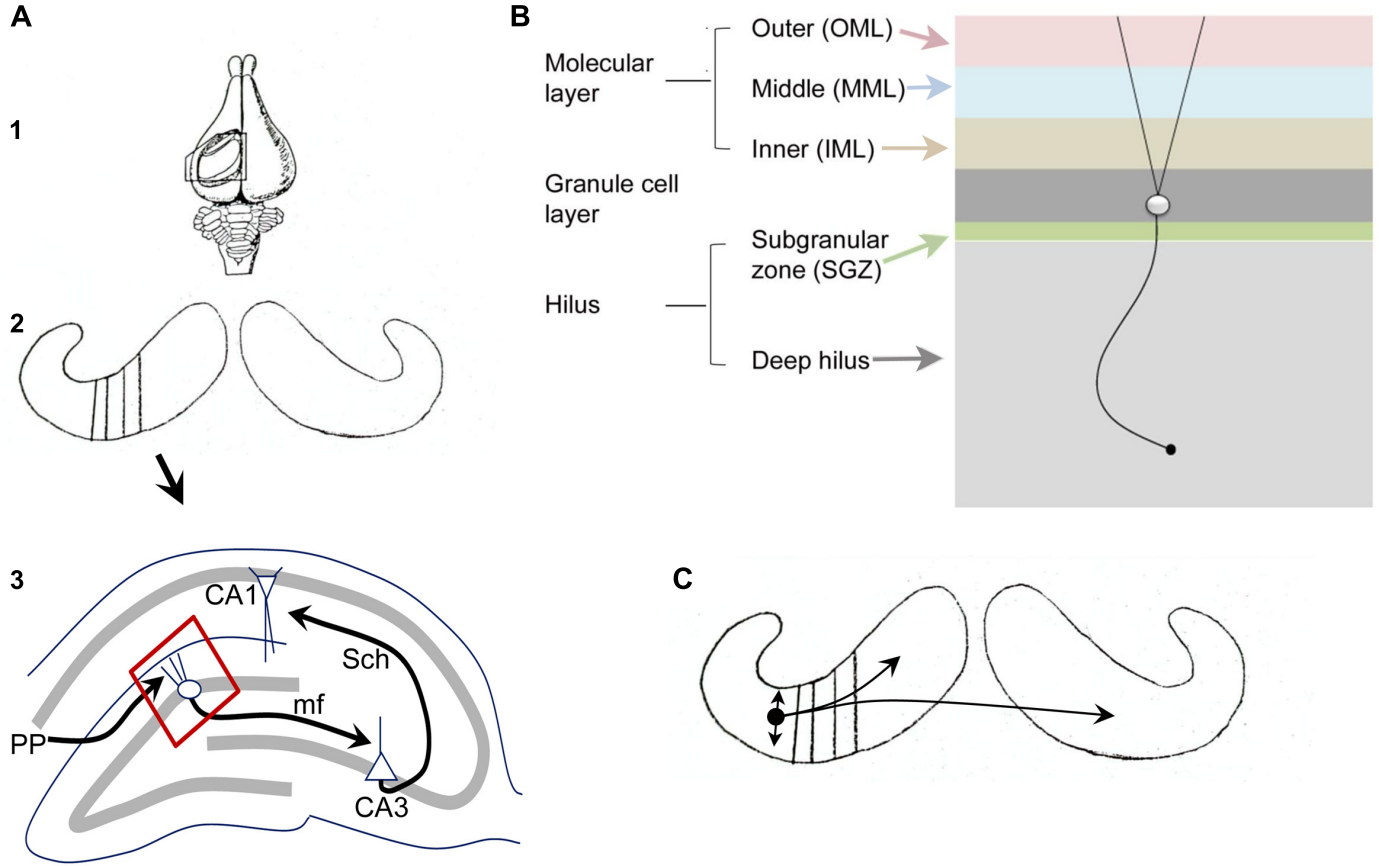
**Fundamental organization of the DG. (A)** (1) A dorsal view of the rabbit brain with the cortex over the hippocampus removed to show the underlying hippocampus. From Andersen et al. (1971). (2) Both hippocampi are shown, with black lines indicating the transverse (lamellar) axis. (3) A single lamella is illustrated to show the trisynaptic circuit. PP, perforant path axons of entorhinal cortical neurons from layer II; mf, mossy fiber axons of granule cells; Sch, Schaffer collateral axons of CA3 pyramidal cells. The area outlined in red is shown in **(B)**. A schematic diagram illustrates the layers of the DG. From (Scharfman and Myers, 2012). **(C)** The schematic from A2 is shown with a small circle indicating the location of a MC body in temporal hippocampus with local projections indicated by the straight arrows, reflecting local collaterals mainly within the hilus, and curved arrows reflecting distant ipsilateral and contralateral projections to the IML. From Scharfman and Myers (2012).

**Figures 1A,B** illustrates the fundamental organization of the dentate gyrus (DG) in rodents (see also Amaral et al., 2007; Scharfman and Myers, 2012). As part of the hippocampal formation, the DG circuitry is organized in repeating units or lamellae, oriented transverse to the long axis of the hippocampus (**Figure 1A**), called the septotemporal axis.

**Figure 1B** illustrates the layers of the DG, which include a molecular layer, granule cell (GC) layer, and hilus. The molecular layer is divided into three subdivisions, the inner, middle, and outer molecular layers. It contains processes of cells in the DG (dendrites, axons) and afferents to the DG, but few cell bodies. The GC layer mainly contains cell bodies of GCs, the primary cell type. The hilus can be divided into a small subgranular zone (SGZ) located just beneath the GC layer, and a larger area called the deep hilus, which ends at the junction with area CA3. The SGZ contains progenitors of GCs that divide periodically throughout life, a process called adult neurogenesis (discussed further below). GABAergic neurons are located in all layers. Mossy cells (MCs, discussed further below) are only located in the hilus.

The major afferent input to the DG is the entorhinal cortical projection from layer II, called the perforant path (PP). The PP terminates in the middle and outer molecular layers and is glutamatergic. There are many additional sources of input to the DG, such as cholinergic and supramammillary fibers to the IML. The hilus contains fibers originating from several extrahippocampal sources, such as cholinergic, noradrenergic, and serotoninergic nuclei. The major projection from the DG is from the GC axons, called mossy fibers, which terminate on hilar neurons and CA3 neurons. Within area CA3, the mossy fibers innervate various GABAergic neurons and the proximal apical dendrites of CA3 pyramidal cells.

Of the cell types in the DG, the GCs, MCs, and GABAergic neurons, the GCs and MCs are relatively homogeneous compared to the diverse types of GABAergic neurons. The GABAergic neurons, often called local circuit neurons or interneurons because they mainly synapse local to the cell body, have somata in all layers, and typically have layer-specific terminal zones. One of the most common subtypes, the so-called basket cell, makes a basket-like plexus around GCs. Basket cells often have a pyramidal cell body located at the base of the GC layer, leading to the term pyramidal basket cell. They are often pooled with another GABAergic neuron subtype that innervates the axon hillock of GCs (axo-axonic cells) to form a subgroup called “perisomatic-targeting” neurons. Neurons located in the hilus that contain the neuropeptides somatostatin (SOM) and/or neuropeptide Y (NPY) make up another major subset of hilar cells and primarily innervate the outer two-thirds of the molecular layer. Another subtype that is important to mention in the context of MCs is a type of GABAergic neuron located in the hilus with an axon that innervates the IML (a GABAergic interneuron with a cell body in the ***hi***lus and a projection to the ***c***ommissural/***a***ssociational ***p***athway, or HICAP cells; Han et al., 1993; Freund and Buzsaki, 1996); it shares a laminar origin (the hilus) and target (the IML) with MCs but is GABAergic and much less numerous than the MCs. Further discussion of the organization of the DG is available elsewhere (Amaral et al., 2007; Scharfman and Myers, 2012).

## Mossy Cells

### The “ABC’s” of MCs

MCs are a major subset of hilar neurons. It has been suggested that they normally comprise 30–40% of neurons in the hilus in the rat (Buckmaster et al., 1996; Jiao and Nadler, 2007).

Based solely on anatomical characteristics, MCs are usually defined by their very large and complex spines on their proximal dendrites, called thorny excrescences. These postsynaptic specializations have multiple postsynaptic densities (PSDs) and oppose the large mossy fiber boutons of GCs. Thorny excrescences also exist on pyramidal cells of CA3, some of which are located deep within the blades of the DG, and some MCs have excrescences that are rather small (Scharfman and Myers, 2012). For these reasons, other characteristics are important to consider when identifying MCs. One characteristic is a large soma, but this criterion needs to be considered cautiously because other hilar GABAergic neurons have large somata and some MCs do not have a large soma (Scharfman and Myers, 2012). MCs can also be distinguished by their axon projection, which primarily targets the IML of the DG, and also has collaterals within the hilus (**Figure 1C**; Scharfman and Myers, 2012). HICAP cells also project to the IML, as mentioned above (Han et al., 1993; Freund and Buzsaki, 1996), but MC axons are distinct from the HICAP cell projections because HICAP projections are local (near the HICAP cell soma, only ipsilateral to the cell body) whereas MCs project far from the MC body to distal hippocampal lamellae, ipsilaterally, and contralaterally, targeting the IML in each case (**Figure 1C**). The projections of MCs are highly organized with respect to the septotemporal axis, with temporal MCs projecting to the septal region of the ipsilateral hippocampus and temporal region of the contralateral DG; conversely, septal MCs project to the temporal pole ipsilaterally and the septal part of the contralateral DG (Blackstad, 1956; Zimmer, 1971; Swanson et al., 1978; Laurberg and Sorensen, 1981; Ribak et al., 1985; Buckmaster et al., 1992, 1996; Wenzel et al., 1997). Other characteristics also distinguish MCs: MCs are regular spiking cells, have very long time constants, a small after hyperpolarization compared to GABAergic neurons, and frequent, large, spontaneous excitatory input (Scharfman and Schwartzkroin, 1988).

In summary, MCs are best defined by a combination of criteria because many of the characteristics have caveats. The one criteria that is an exception, being unique to MCs and a defining characteristic of all MCs, is the axon projection.

### Excitatory and Inhibitory Effects of MCs

MCs primarily use glutamate as a neurotransmitter (Soriano and Frotscher, 1994; Scharfman, 1995; Wenzel et al., 1997). In this regard, they are similar to principal cells. Consistent with this view, their intrinsic properties are similar to glutamatergic regular spiking cells, not GABAergic fast spiking cells (Scharfman and Schwartzkroin, 1988; Scharfman, 1992; Lubke et al., 1998). Because MCs make monosynaptic connections onto GCs, one would predict that MCs would depolarize GCs and lead to action potential generation. This idea is supported, where it has been possible to record from pairs of monosynaptically connected MCs and GCs. Thus, if one records from synaptically coupled MCs and GCs simultaneously in hippocampal slices, short latency (consistent with a monosynaptic pathway) depolarizations can be recorded in GCs in response to selective excitation of the MC (Scharfman, 1995). Furthermore, when MCs in hippocampal slices are activated by channelrhodopsin, short-latency EPSCs are recorded in GCs (Chancey et al., 2014).

However, the unitary EPSP of the MC–GC synapse appears to be weak, at least at the resting potential of GCs, which is more hyperpolarized than other DG and hippocampal neurons (Scharfman, 1995). Moreover, MCs depolarize monosynaptically coupled interneurons in the hilus (Scharfman, 1995; Larimer and Strowbridge, 2008). Therefore, it is not clear how ‘excitatory’ MCs actually are. Notably, some DG inhibitory neurons have commissural connections, such as a subset of SOM- and parvalbumin (PV)-expressing GABAergic neurons (Bakst et al., 1986; Goodman and Sloviter, 1992; Deller et al., 1995). Therefore, MCs could have disynaptic inhibitory effects that are not only local but bilateral, and these effects could be as strong as the excitatory effects of MCs.

For these reasons, it has been unclear whether the net effect of MCs is excitatory or inhibitory. In other words, will excitation of MCs lead to an increase or a decrease in GC output to CA3? Buckmaster and Schwartzkroin (1994) proposed that MCs ordinarily excite GCs and this leads to an associational effect. There are several studies that support this hypothesis. For example, deletion of MCs in slices led to reduced excitation rather than increased excitation (Ratzliff et al., 2004). Extensive quantification of MC synapses on distal GCs showed preferential innervation of GCs rather than interneurons (Buckmaster et al., 1996).

Another hypothesis is that MCs primarily excite interneurons such as basket cells (Sloviter, 1991, 1994; Sloviter et al., 2003). This idea emerged from *in vivo* recordings of GCs using extracellular recording methods in anesthetized rats. MCs were killed by intermittent stimulation of the PP, probably due to excess glutamate release from strongly activated ‘giant’ boutons of mossy fibers releasing high concentrations of glutamate on MCs (Sloviter, 1983). The recordings that were made *in vivo* after MC loss showed greater activation of GCs by electrical stimulation of the PP (Sloviter, 1991). Stimulation of the PP could elicit multiple, synchronized action potentials in the GC population near the recording electrode, or “population spikes,” which indicates hyperexcitability of the GCs. Simulating the intermittent stimulation in hippocampal slices had a similar effect (Scharfman and Schwartzkroin, 1990b). In addition, slice recordings demonstrated that spontaneous burst discharges could be recorded in area CA3 after intermittent stimulation, another indication of hyperexcitability (Scharfman and Schwartzkroin, 1990a). Traumatic brain injury (TBI), which also causes MC loss, also led to multiple population spikes in the DG in response to PP stimulation *in vivo* (Lowenstein et al., 1992) and *in vitro* (Santhakumar et al., 2001). Additional support for this hypothesis was provided from a study of transgenic mice where MCs were deleted and multiple population spikes developed in response to electrical stimulation of the PP (discussed further below; Jinde et al., 2012). Together these experiments suggested that MCs normally activated GABAergic interneurons that in turn inhibited GCs from firing action potentials. Basket cells were implicated because they are one of the most common GABAergic cell types in the DG, and inhibit GC action potential generation by axon terminals that surround the cell body (see discussion in Sloviter, 1991, 1994; Sloviter et al., 2003). However, there are arguments against the hypothesis (e.g., Bernard et al., 1998).

A combination of the two hypotheses has also been suggested, based on the rich collateralization of MCs in the hilus near the MC soma (Scharfman and Schwartzkroin, 1988; Scharfman and Myers, 2012) and recordings showing that MCs depolarize their hilar interneuron targets in the vicinity of the MC soma (Scharfman, 1995; Larimer and Strowbridge, 2008). The idea that MCs might activate interneurons locally but excite GCs distally was called the ‘integrative’ hypothesis (Scharfman and Myers, 2012). One reason to suggest the hypothesis was based on results from additional recordings in hippocampal slices: there were excitatory effects of MCs on monosynaptically coupled GCs in slices (i.e., GCs close to the MC body) that were only possible to detect when GABAergic inhibition was blocked (Scharfman, 1994a,b).

The debate regarding excitatory vs. inhibitory effects of MCs continues as more data and more experimental approaches are used. For example, excitatory effects of MCs on GCs, without blockade of GABA receptors, has been shown in slices (i.e., in the vicinity of the MC soma) using voltage imaging and optogenetic methods (Jackson and Scharfman, 1996; Chancey et al., 2014; Wright and Jackson, 2014). Therefore, MCs may have robust excitatory connectivity with GCs near the MC soma, arguing against the integrative hypothesis. It has also been suggested that there are subtypes of MCs, based on morphologyical and physiological criteria (Scharfman and Myers, 2012) as well as c-fos immunoreactivity (discussed further below; Duffy et al., 2013), which makes the interpretation of previously published data more complex.

### The Potential Roles of MCs in Behavior

Although a great deal is known about MCs in terms of their morphology and connectivity, there are almost no studies of MCs in behavior. However, laboratories have studied MCs during theta oscillations *in vivo*, so one can relate some data about MCs to the behaviors associated with theta rhythm. One study found that MCs discharge similar to other hippocampal principal cells during the peaks of theta oscillations (Soltesz et al., 1993). Therefore, MCs are likely to contribute to the hippocampal functions that are related to theta, such as spatial navigation and spatial memory, although exactly how is unclear. One possibility, suggested from the recordings *in vivo*, is that MCs synchronize or “phase-lock” DG neurons because of their axonal projections to many GCs across the septotemporal axis and contralateral DG (Soltesz et al., 1993). MCs have also been discussed in the context of sharp waves recorded *in vivo*, where MCs discharge like other principal cells (discussed in Penttonen et al., 1997), implicating MCs in sharp wave-related phenomena (i.e., hippocampal-dependent memory consolidation). More recently it was suggested that MCs may have spatial firing patterns with multiple place fields that are distinct from GCs, suggesting that MCs play a unique role in the transformation of entorhinal input within the DG (Neunuebel and Knierim, 2012).

C-fos, an immediate early gene, has also been a tool to understand MCs *in vivo* because antibodies to c-fos mark cells that have been active in the hours prior to perfusion-fixation. Remarkably, a subset of MCs were labeled by antibodies to c-fos even if animals were not engaged in a behavioral task prior to euthanasia (Duffy et al., 2013). These data suggest that some MCs are active without a behavioral challenge. Interestingly, acclimation to the surroundings decreased MC c-fos expression, suggesting that MCs are activated by novelty in their environment (Duffy et al., 2013). Therefore, MCs may play a role in the circuitry that underlies novelty recognition or its memory.

Elegant electrophysiological studies have also suggested that MCs can “distinguish stimuli” with a different approach; these experiments focused on circuitry between the semilunar GCs, MCs, and GABAergic neurons in the hilus of hippocampal slices. Semilunar GCs are a type of GC located in the IML that have very large, prolonged depolarizations to PP stimulation and activate MCs as well as hilar GABAergic neurons (Williams et al., 2007; Larimer and Strowbridge, 2008, 2010; Gupta et al., 2012). The recordings suggested that the local circuitry could allow different PP stimuli to be discriminated (Larimer and Strowbridge, 2008, 2010). It was also proposed that the prolonged depolarizations of semilunar GCs, which are not characteristic of other GCs, caused prolonged activity in hilar neurons creating hilar “up states” (Larimer and Strowbridge, 2008, 2010). That finding is potentially very important because of the role of cortical up states in working memory; the data suggest that hilar cells, including MCs, play an important role in working memory because they have up states in response to cortical input (discussed further in Larimer and Strowbridge, 2008, 2010).

MC-specific targeting in transgenic mice has played an important role in understanding how MCs are involved in behavior. A transgenic mouse was used that took advantage of a marker specific to MCs, calcitonin receptor-like receptor (CRLR). CRLR-Cre transgenic mice were generated and crossed to mice with a floxed diphtheria toxin receptor gene; administration of diphtheria toxin to the offspring of these mice resulted in deletion of MCs. After deletion of MCs, mice showed impairments in a contextual discrimination task, which depends on the DG. Mice also displayed behavior consistent with increased anxiety in open field, elevated–plus and forced swim tasks. In addition, recordings showed multiple population spikes (hyperexcitability) of GCs in response to electrical stimulation of the PP (Jinde et al., 2012). This study was the first to directly implicate MCs in DG-dependent behaviors *in vivo*. However, there were limitations, such as selectivity: a subset of CA3 neurons were deleted as well as MCs. Moreover, behavioral and physiological effects were transient, resolving after 4–6 weeks, which was attributed to compensatory sprouting of interneurons in the DG (Jinde et al., 2012).

In summary, there is little information about MCs during behavior *in vivo*. However, the data that are available suggest – as one might expect – that MCs are important to many normal behavioral functions of the hippocampus.

### Hypotheses about MCs in Disease

#### Psychiatric Disorders

Recent studies have implicated MCs not only in normal DG functions, but also in psychiatric disease, specifically anxiety, depression, and schizophrenia. The evidence that MCs regulate anxiety comes primarily from studies of their deletion (Jinde et al., 2012). Regarding depression, MCs have been discussed in the context of the proteins p11 and SMARCA3, which have recently been shown to play a critical role in the actions of the class of antidepressants that increase serotonin levels (selective serotonin reuptake inhibitors; SSRIs). To demonstrate this role, transgenic mice were used with enhanced green fluorescent protein (EGFP) coexpressed in cells where p11 was localized, which revealed that p11 is enriched in MCs and basket cells (Oh et al., 2013). Additionally, treatment with the SSRI fluoxetine increased expression of p11 and SMARCA3 in MCs and basket cells (Oh et al., 2013). Moreover, fluoxetine increased adult neurogenesis in wild type mice, but SMARCA3 knockout (KO) mice were unaffected, and the KO mice showed no behavioral response to antidepressant administration (Oh et al., 2013). A similar effect was also shown in p11 KO mice (Egeland et al., 2010). Therefore, SMARCA3 and p11 appear to be necessary for the increase in adult neurogenesis mediated by SSRIs and the improved behavioral responses of mice treated with SSRIs. The specific role of MCs was not possible to address, however, because p11 and SMARCA3 are expressed in both MCs and basket cells.

MCs have also been discussed in the context of schizophrenia. These studies focus on dysbindin-1, which is involved in many functions of neurons, including synaptic transmission, protein trafficking, and lysosomal pathways. Dysbindin-1 is decreased in the hippocampus in patients with schizophrenia (Talbot et al., 2011), and – within the hippocampus – is localized to MCs (Wang et al., 2014). Notably, mice that lacked dysbindin-1C (one of three isoforms of dysbindin-1) had fewer MCs. Interestingly, the mice with reduced numbers of MCs showed a delay in maturation of newborn GCs (Wang et al., 2014), supporting a role of MCs in trophic support of adult-born GCs (discussed further below). A later study suggested that dysbindin-1C deletion caused a reduction in MCs by interfering with the normal pathways for clearing waste from neurons (i.e., autophagy; Yuan et al., 2015). However, the association between MCs, dysbindin-1C, and schizophrenia remains unclear.

#### Seizures and Epilepsy

MCs were first implicated in temporal lobe epilepsy (TLE) after it was identified that hilar neurons were typically lost in neuropathological specimens from patients that had TLE (Margerison and Corsellis, 1966). The name for the particular pattern of hilar cell loss observed in TLE is “endfolium sclerosis” where ‘endfolium’ refers to the hilus (Scharfman and Pedley, 2006). It subsequently became clear that many patients with TLE show additional damage in other hippocampal regions (e.g., CA1, CA3) and the term mesial temporal lobe sclerosis (MTS) is now used to refer to the more extensive pattern of neuronal loss (Scharfman and Pedley, 2006).

After hilar neuron subtypes were identified and experimental models of TLE were developed, investigators began to study the role of MC loss in TLE. It was hypothesized that MC loss caused epilepsy, either because MCs were critical to activate inhibitory neurons of the DG (the “dormant basket cell” hypothesis; Sloviter, 1991, 1994; Sloviter et al., 2003), or because residual MCs with increased excitability activated the GCs (the irritable MC hypothesis; Santhakumar et al., 2000; Ratzliff et al., 2002 and for additional discussion see Scharfman and Myers, 2012). Importantly, the one study published to date that induced selective deletion of MCs did not lead to a TLE-like syndrome. However, transient hyperexcitability was demonstrated, as mentioned above (Jinde et al., 2012).

In summary, initial hypotheses about MCs in disease have emerged in recent years, and those that have been developing for decades are being tested by more selective techniques using mice with Cre recombinase preferentially in MCs. However, the role of MCs in psychiatric disease remains mostly speculative and the role of MCs in TLE is still debated.

## Adult Neurogenesis in the DG

In most of the initial studies of MCs, the potential role of adult-born GCs in the circuitry that involves MCs was not considered because adult neurogenesis in the DG was not widely accepted. Therefore, it is timely to consider adult neurogenesis now.

### The Role of Adult-Born Neurons in DG-Dependent Functions

After it was accepted that neurogenesis occurred in the adult mammalian brain, it was not clear that adult neurogenesis in the DG would be important because adult-born GCs do not give rise to more than a few percent of the GCs at any given time (Cameron and McKay, 2001). However, it is now clear that the small population of adult-born GCs exerts an important influence on the DG. Adult-born neurons have been shown to play a role in numerous DG-dependent behaviors, including pattern separation and contextual fear conditioning (Saxe et al., 2006; Winocur et al., 2006; Clelland et al., 2009; Hernandez-Rabaza et al., 2009; Denny et al., 2012; Nakashiba et al., 2012; Niibori et al., 2012). Other behaviors that involve the DG are also affected, such as cognitive flexibility (Burghardt et al., 2012) and detection of novelty (Jessberger et al., 2009; Denny et al., 2012).

How do adult-born GCs exert these functions? It has been suggested that they are highly excitable when they are young, based on direct electrophysiological recordings in hippocampal slices. For example, immature adult-born neurons have a higher input resistance than mature GCs (Ambrogini et al., 2004; Esposito et al., 2005; Dieni et al., 2013), and exhibit a depolarizing rather than hyperpolarizing response to GABA (Markwardt and Overstreet-Wadiche, 2008) as well as other unique characteristics that increase the ratio of excitation to inhibition (Marin-Burgin et al., 2012). Young adult-born GCs also have greater plasticity than mature GCs (Snyder et al., 2001; Schmidt-Hieber et al., 2004; Gu et al., 2012). As a result of the addition of more excitable and more plastic cells to the GC population, one would predict improved function.

However, more excitation in the DG may not improve its function, and young adult-born GCs may inhibit mature GCs, not excite them. At the circuit level, there are currently two hypotheses: (1) young adult-born GCs preferentially excite mature GCs and area CA3, and (2) young adult-born GCs preferentially inhibit mature GCs, and therefore the main output of the DG to area CA3 is inhibited.

The idea that adult-born GCs might inhibit mature GCs emerged from the idea that GCs discharge rarely or sparsely *in vivo* (Jung and McNaughton, 1993), and the inhibition of the GCs is necessary to maintain a network that can perform pattern separation (Marr, 1971; McNaughton and Morris, 1987; Rolls, 2013; Kesner and Rolls, 2015). In this context, pattern separation refers to the ability to separate overlapping inputs, so the pattern of output from the network is less overlapping than the pattern of input. Investigators found that mice with suppressed adult neurogenesis performed relatively poorly in tasks that were intended to test pattern separation (Clelland et al., 2009), and behavior improved when adult neurogenesis was selectively increased (Sahay et al., 2011). Therefore, it was suggested that increased inhibition of the mature GCs by adult-born GCs might be one mechanism that supports pattern separation (Aimone et al., 2011; Sahay et al., 2011).

Other studies suggested that adult-born GCs increased inhibitory tone in the DG. For example, when adult-born GCs were ablated by focal X-irradiation, spontaneous bursts of GC activity increased in the DG (Lacefield et al., 2012) consistent with a disinhibitory effect. When animals with and without adult-born neurons were compared, mice with reduced adult neurogenesis showed increased expression of the immediate early gene Arc after a behavioral task that assessed cognitive flexibility (Burghardt et al., 2012). In addition, slices of the DG with reduced numbers of adult-born GCs showed greater spread of activity within the DG in response to a PP stimulus (Ikrar et al., 2013). One potential caveat with these studies was that ablation of adult-born GCs may have induced compensatory changes that increased excitability, such as sprouting of the supramammillary input (Singer et al., 2011). However, optogenetic activation of young adult-GCs evoked primarily inhibitory synaptic currents (IPSCs) in mature GCs, relative to excitatory synaptic currents (EPSCs; Drew et al., 2013).

The argument that adult-born GCs do not enhance inhibition in the DG comes from studies showing that they support LTP as discussed above; typically inhibition does not enhance LTP – disinhibition does (Arima-Yoshida et al., 2011). In addition, a recent study using optogenetics to activate 4 week-old GCs showed weak feedback inhibition of mature GCs rather than stronger feedback inhibition (Temprana et al., 2015). More experiments will be necessary to clarify why some studies show that adult-born GCs increase DG inhibition and others do not. The conditions used for animals (e.g., enriched environment or not) as well as other experimental variables (e.g., age, sex) could be important to the effects of adult-born GCs on DG inhibition (discussed further below).

### The Role of Adult-Born Neurons in Psychiatric Disorders

Adult-born neurons have been implicated in mood regulation and depression (Sahay et al., 2007). A role of adult-born neurons was first hypothesized when several antidepressant medications lost their efficacy in mice with depletion of adult-born neurons by focal X-irradiation (Santarelli et al., 2003). This led to the idea that adult-born neurons are required for the antidepressant effect, and reducing the numbers of adult-born neurons or impairing their function might cause depression.

Adult neurogenesis has also been implicated in a familial form of schizophrenia where there is a disrupted-in-schizophrenia (DISC1) mutation, leading to abnormal maturation of adult-born GCs (Duan et al., 2007). Other rodent models of schizophrenia such as 22q11.2 deletion-associated schizophrenia, suggest a role of adult-born GCs also (Toro and Deakin, 2007; Ouchi et al., 2013).

The potential role of adult-born GCs in depression and schizophrenia is interesting in light of the evidence that MCs could also play a role in these two disorders. This similarity could be due to the fact that MCs exert some of their effect on the DG via excitation of adult-born GCs.

### Adult-Born Neurons in Seizures and Epilepsy

#### Seizures and Adult Neurogenesis

There is a great deal that is known about adult DG neurogenesis in the context of seizures. Regarding a single episode of experimentally induced seizures, it was shown in 1997 that proliferation greatly increases after a single afterdischarge (Bengzon et al., 1997) or status epilepticus (SE; Parent et al., 1997) and subsequently this result was reproduced in numerous laboratories with other seizure-induction protocols (for review, see; Scharfman and McCloskey, 2009). What is rarely studied is whether normal adult-born GCs regulate seizure activity. One reason may be that it seems unlikely that normal adult-born GCs would affect seizures because it is hard to conceive of a robust effect of such a small population of cells on a seizure, in which so many neurons are affected.

**FIGURE 2:**
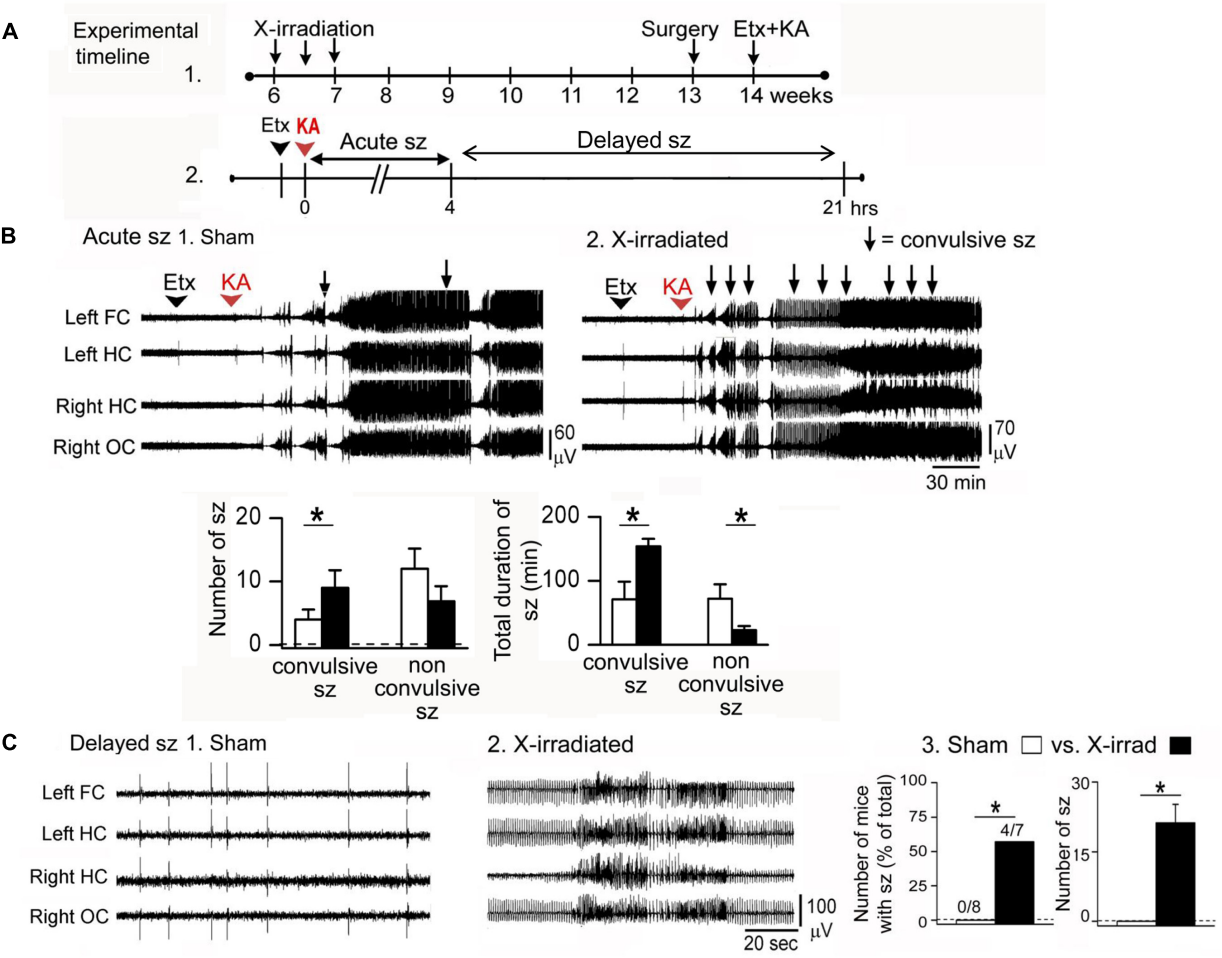
**Ablation of adult-born GCs leads to an increased effect of the convulsant kainic acid. (A)** (1) Effects of kainic acid (KA) were tested in mice that had three sessions of focal X-irradiation and then were implanted with electrodes. After 1 week for recovery from electrode implantation, mice were injected with KA. (2) Effects of KA were characterized as ‘acute’ (Acute sz), referring to the first 4 h after injection when seizures were severe and occurred with brief interseizure intervals. Effects of KA also were examined after this acute period, when intermittent interictal spiking occurred. In some cases there were seizures, which are referred to as delayed seizures (Delayed sz). **(B)** In the acute period after KA injection, mice with reduced adult neurogenesis had more convulsive seizures and the total duration of all convulsive seizures was increased compared to sham-irradiated mice. There were fewer non-convulsive seizures, presumably because many of these seizures became convulsive in mice with reduced adult neurogenesis. **(C)** Delayed seizures are compared. (1) A typical period of interictal spiking in a sham-irradiated mouse, over 4 h after KA injection. (2) A typical record from an X-irradiated mouse at approximately the same time after KA injection shows more intense spiking and a seizure. (3) Delayed seizures did not occur in mice with reduced adult neurogenesis but they did occur in mice with focal X-irradiation. Left: number of mice with delayed seizures compared to all mice tested. Right: number of seizures in sham and X-irradiated mice. Asterisks denote significance (*p* < 0.05). Note that focal X-irradiated mice were similar to other mice with suppressed neurogenesis using other methods to reduce adult neurogenesis. From Iyengar et al. (2015).

However, experiments using kainic acid-induced seizures support the hypothesis that normal adult-born GCs inhibit seizures (Iyengar et al., 2015; **Figure 2**). In that study, focal low-dose X-irradiation and a pharmacogenetic approach were used to reduce adult-born GCs. X-irradiated mice were administered kainic acid 7 weeks after X-irradiation to ensure that all effects of the irradiation procedure were over. The pharmacogenetic approach used mice with thymidine kinase (TK) in GFAP-expressing neurons (GFAP-TK). Mice were fed valganciclovir 5 days/week for 6 weeks to activate TK in dividing GFAP-expressing progenitors. One week after the valganciclovir treatment ended, 7 weeks after the onset of treatment, animals were implanted with electrodes, and 1 week later they were injected systemically with kainic acid.

Two procedures were used: either the T-type calcium channel antagonist ethosuximide was administered, followed 30 min later by kainic acid, or kainic acid alone. Use of ethosuximide appears to suppress brainstem seizure activity which normally dominates the response to kainic acid. With this procedure, seizures could be examined that involved forebrain structures and therefore would be potentially influenced by adult neurogenesis.

The animals that had reduced adult neurogenesis had a more severe (convulsive) effect of kainic acid, especially when ethosuximide was used. Latency to the first convulsive seizure was decreased, and the number of convulsive seizures and their duration were increased when adult-born GCs were reduced (Iyengar et al., 2015). Unexpectedly, there was a greater effect in GFAP-TK mice compared to X-irradiated mice. One reason could be that GFAP-TK mice had reduced adult neurogenesis in both the DG and olfactory bulb but X-irradiated mice had reduced adult-born neurons only in the DG, and both the DG and olfactory bulb have been shown to inhibit seizures (Iyengar et al., 2015).

The fact that adult neurogenesis influenced convulsive seizures preferentially is interesting because it suggests that a small number of neurons could exert a dramatic effect *in vivo.* In addition, the experiments support the view that adult-born GCs – up to about 7 weeks of age – normally are inhibitory in their effects.

How are the results reconciled with the studies of Temprana et al. (2015) who suggested that adult-born GCs do not exert a strong effect on feedback inhibition of GCs? One possibility is that Temprana et al. (2015) studied animals under different conditions. Notably, if the environment of developing adult-born GCs is enriched, the circuitry of those GCs develops very differently (Bergami et al., 2015). Another idea is that adult-born GCs produce a more persistent inhibition than mature GCs despite weak inhibition in response to a single stimulus, which was suggested (Temprana et al., 2015). Thus, even though feedback inhibition was weak when examined after a single, isolated stimulus, it was noted that there was stronger inhibition in response to stimulus trains (Temprana et al., 2015). The reason this point is potentially relevant is that in a seizure there is a persistent train of firing of presynaptic afferents, not an isolated presynaptic stimulus. Therefore, during a seizure, adult-born GCs may keep the seizure activity in the DG from being extremely severe.

#### Adult-Born Neurons in TLE

Distinct from acute seizures in the normal brain is epilepsy, where seizures are spontaneous, recurrent, and associated with neuropathology. In TLE, the pathology includes various degrees of altered gene expression, circuitry, glia, and vasculature. In animal models of acquired TLE, where these pathological changes are extensive, adult-neurogenesis has been studied by many laboratories.

**FIGURE 3:**
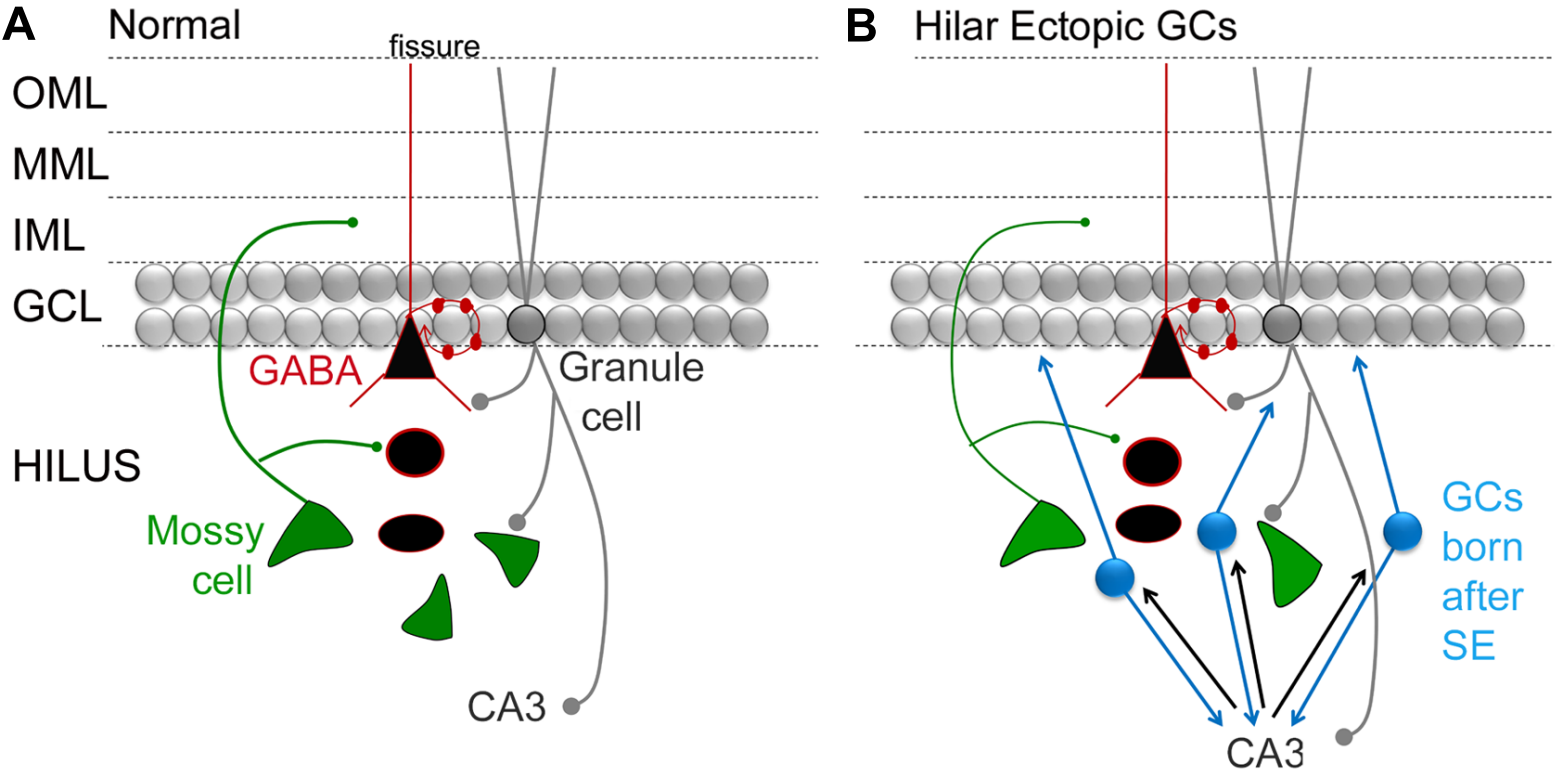
**Circuitry in the DG that involves MCs and adult-born GCs. (A)** A simplified view of DG circuitry shows major pathways of MCs (green), perisomatic targeting cells (e.g., basket cells; black triangle outlined in red) and granule cells (gray circles). MCs innervate local GABAergic neurons with dendrites in the hilus. They also innervate GC proximal dendrites in the IML. Basket cells form a basket like axon plexus around GC somata. GCs innervate hilar neurons and area CA3 neurons. OML, outer molecular layer; MML, middle molecular layer; IML, inner molecular layer; GCL, granule cell layer. **(B)** A schematic that shows the DG circuitry after severe experimental seizures (status epilepticus), where a population of GCs develops in the hilar region (hilar ectopic GCs, blue). These GCs have mossy fiber axons that synapse in CA3, like normal mossy fibers, and in the IML, contributing to mossy fiber sprouting. The resultant circuitry potentially becomes a focus for seizures, because reducing the hilar ectopic GC population decreases seizures (see text for further discussion).

In many of these animals, SE is induced by administration of a convulsant in adult rodents, and a TLE-like syndrome develops in the subsequent weeks. It has been shown that there is a surge in proliferation in the days after SE (Parent et al., 1997). Diverse abnormalities have been described in the GCs born after SE: some neurons develop altered structure, location, and orientation (Scharfman et al., 2007; Shapiro et al., 2008; Murphy et al., 2012). The neurons that are in an abnormal location are often in the hilus rather than the GC layer, and these hilar ‘ectopic’ GCs (hEGCs) develop abnormal connections that foster recurrent excitation (**Figure 3**; Scharfman, 2004; Scharfman and Pierce, 2012). For these reasons, the hEGCs were originally suggested to promote rather than inhibit seizures (Scharfman et al., 2000; Scharfman, 2004). Subsequently there was support for this hypothesis from studies which used unselective inhibition of adult neurogenesis such as the mitotic inhibitors (Jung et al., 2004). In this study, cytosine-b-D-arabinofuranoside administration after SE led to fewer hEGCs and fewer spontaneous recurrent seizures 4 weeks after SE (Jung et al., 2004). These studies ultimately were followed by more specific approaches such as selective ablation of nestin-expressing precursors, which also reduced hEGCs and chronic seizures after SE (Cho et al., 2015). The converse also appears to be true, i.e., increasing aberrant neurogenesis increases seizures. In the one study showing this effect, Pun et al. (2012) studied abnormal new GCs that were hypertrophied rather than ectopic. Initiating the hypertrophy led to spontaneous seizures. The results suggest that aberrant neurons may have adverse effects, and that diverse types of abnormalities (hypertrophy, ectopic cells) can have adverse consequences. However, there are studies that do not find an effect of aberrant neurogenesis on seizures in epilepsy models (Pekcec et al., 2008, 2011; Kondratiuk et al., 2015). On the other hand, differences in methods and animal models could explain the discrepancies. Therefore, more research will be necessary to clarify the conditions where acute seizures and chronic epilepsy is affected by adult neurogenesis.

## The MC → Adult-Born GC Pathway

### Evidence that MCs Innervate Adult-Born GCs

Several laboratories have now shown that MCs innervate adult-born GCs (Kumamoto et al., 2012; Vivar et al., 2012; Deshpande et al., 2013; Chancey et al., 2014). The synapses between MCs and adult-born GCs are the first glutamatergic synapses of adult-born GCs (Deshpande et al., 2013; Chancey et al., 2014), forming approximately 2 weeks after birth, about 1 week before the synapses between the medial or lateral PP and adult-born GCs (Kumamoto et al., 2012; Vivar et al., 2012; Deshpande et al., 2013). Therefore, the first afferents to adult-born GCs come from GABAergic neurons and MCs. However, there are additional inputs in these first few weeks: cholinergic neurons from the septum and nucleus of the diagonal band, and area CA3 neurons provide afferent input; mature GCs also innervate adult-born GCs, although only transiently (Vivar et al., 2012; Deshpande et al., 2013).

There are some potential pitfalls with the rabies virus approach used for some of these studies (as discussed in Chancey et al., 2014) so it was very important to show electrophysiological evidence that the MC → adult-born GC synapse is functionally excitatory (Chancey et al., 2014). Notably, the EPSC caused by MC activation appears to be small, approximately 5 pA compared to >20 pA for the the PP input (Chancey et al., 2014). However, the input from MCs is proximal to the GC cell body compared to the PP input, and young GCs have higher input resistance than older GCs (Mongiat et al., 2009). Therefore, a small EPSC in young GCs that is generated proximal to the soma may have a robust effect.

The EPSC may also be effective when timed so that it occurs in conjunction with another excitatory input, which was previously suggested when the MC → mature GC synapse was first characterized (Scharfman, 1995). That synapse had greater excitatory effects when the postsynaptic GC was artificially depolarized by tonic current from the recording electrode (Scharfman, 1995). Therefore it is interesting to consider what other depolarizing inputs to adult-born GCs are present. In the adult IML, there are inputs from the supramammillary nucleus (Wyss et al., 1979; Stanfield et al., 1980; Carre and Harley, 1991; Nakanishi et al., 2001) as well as cholinergic input (Green and Mesulam, 1988; Kitt et al., 1994) but whether these inputs are on adult-born GCs or mature GCs is not known. GABAergic inputs to the adult-born GCs could have an excitatory influence when adult-born GCs are young because effects of GABA depolarize rather than hyperpolarize the young GCs (Dieni et al., 2012; Chancey et al., 2013). However, whether there is a synergism with the MC input, or a decrease in efficacy because of “shunting” inhibition is not clear.

### Implications of the MC → Adult-Born GC Synapse

There are several potential functions of the MC → adult-born GC synapse. One is developmental: MCs may stimulate the developing GC by release of glutamate from terminals in the IML. The only evidence for this hypothesis is mentioned above, the study of Wang et al. (2014), where mice with fewer MCs had fewer adult-born GCs. However, there was no proof of direct effects of MC loss on adult-born GCs. In addition, Jinde et al. (2012) did not detect changes in staining of adult-born GCs after MC deletion, using antibodies for doublecortin, a marker of immature neurons.

A second hypothesis is synaptic: MCs could provide an important source of excitatory synaptic input to adult-born GCs that is critical to information processing in the DG. Given the functional evidence that the MC adult-born GC synapse is strong (Chancey et al., 2014), we consider the implications of the second hypothesis below.

#### The MC → Adult-Born GC Synapse in Normal Conditions

**FIGURE 4:**
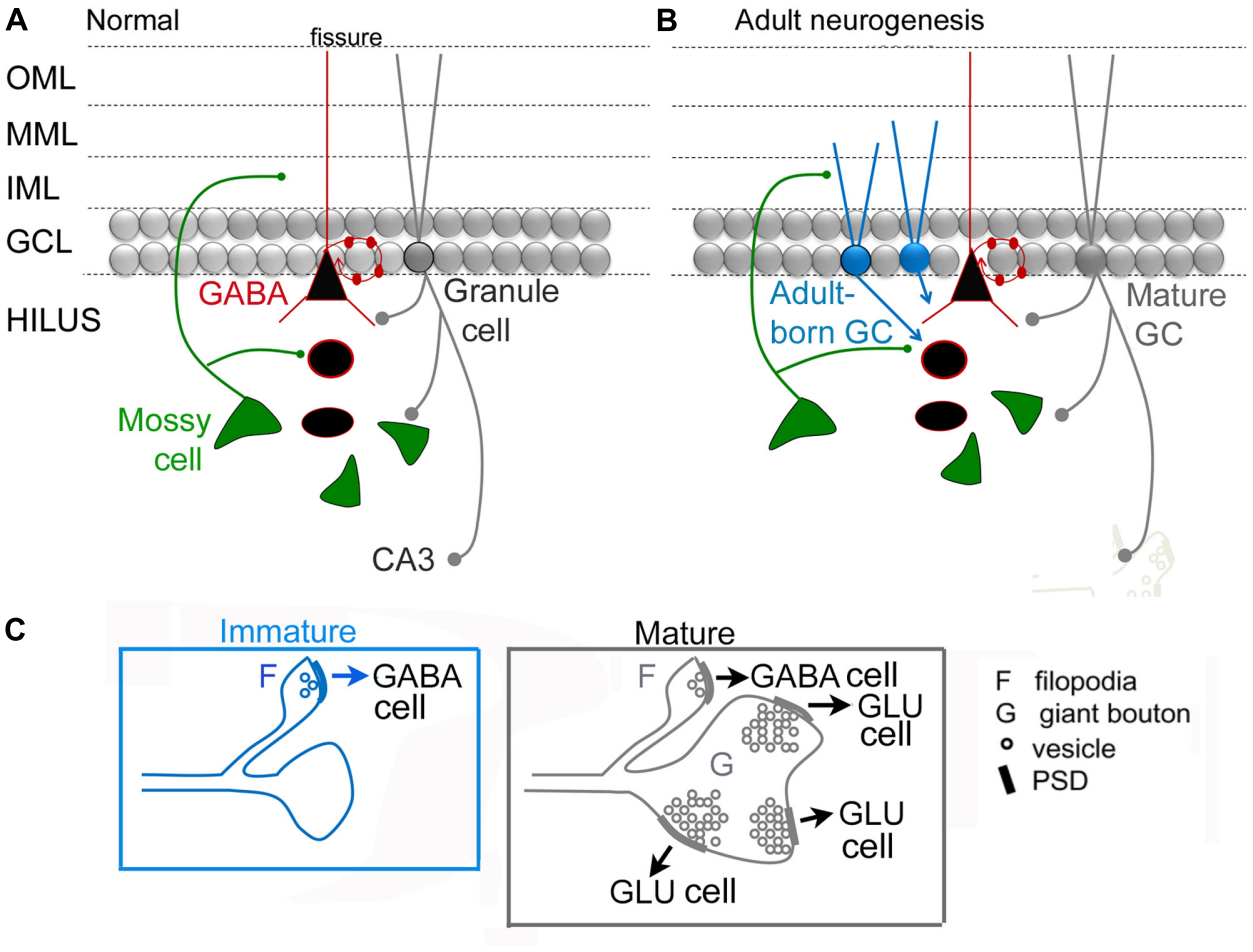
**Dentate gyrus circuitry with and without adult-born GCs. (A,B)**. A previous **(A)** and updated **(B)** view of DG circuitry with a young adult-born GC (blue) depicted at an immature stage of development when their dendrites extend only a short distance into the molecular layer. MCs are the primary glutamatergic input at this time. Adult-born cells are not completely mature and their axon preferentially innervates GABAergic neurons. At a mature stage they develop more of their “giant” boutons which innervate thorny excrescences of mossy and pyramidal cells. We hypothesize that the adult-born GCs primarily innervate GABAergic interneurons when the adult-born GC is young because ablation of adult-born GCs has a proconvulsant effect (**Figure 2**). **(C)** Comparison of an immature GC axon with filipodia (F) that have postsynaptic densities (PSDs) with synaptic vesicles (o) which contact GABAergic neurons primarily. These data are from studies of GCs born in early life (for discussion and references, see text). After several weeks of age, these GC axons have filipodial extensions that contact GABAergic neurons and giant boutons (G) with multiple postsynaptic densities (PSDs) and numerous synaptic vesicles (o) that mainly contact principal cells (GLU).

**Figure 4** shows a circuit diagram of the DG that reflects prior views (**Figure 4A**) and a new view (**Figure 4B**) with the MC → young adult-born GC synapse added (**Figure 4B**). A critical element of this circuitry is exactly what neurons adult-born GCs innervate. It has been shown that adult-born GCs, once they are mature, excite hilar neurons (interneurons and MCs) and area CA3 pyramidal cells (Toni et al., 2008). These data suggest that the neurons innervated by adult-born GCs are similar to those targeted by GCs born in early life (or mature GCs born in adulthood), although quantitative comparisons of these three types of GCs have not yet been made. Young, adult-born GCs could be different from GCs that are born in early life because as GCs develop in early life there initially are small varicosities on the mossy fiber axon before the giant boutons emerge (Amaral and Dent, 1981). Interestingly, mossy fibers continue to mature for weeks in GCs born in early life (Stirling and Bliss, 1978; Amaral and Dent, 1981). In the rat, mossy fibers of these GCs have small terminals in the hilus initially, and larger terminals are restricted to area CA3 (Ribak and Navetta, 1994). Small varicosities from extensions of mossy fibers are common at this early age (Amaral, 1978; **Figure 4C**). The small varicosities appear to innervate interneurons primarily, at least when mossy fibers are studied in adulthood (Acsady et al., 1998) whereas the larger boutons innervate thorny excrescences which are only on MCs and PCs (Blackstad and Kjaerheim, 1961; Amaral, 1978; Chicurel and Harris, 1992; Acsady et al., 1998; Scharfman and Myers, 2012; **Figure 4C**). Therefore, in early stages of development, adult-born GCs could be primarily inhibitory and then both inhibitory and excitatory (**Figure 4C**).

**FIGURE 5:**
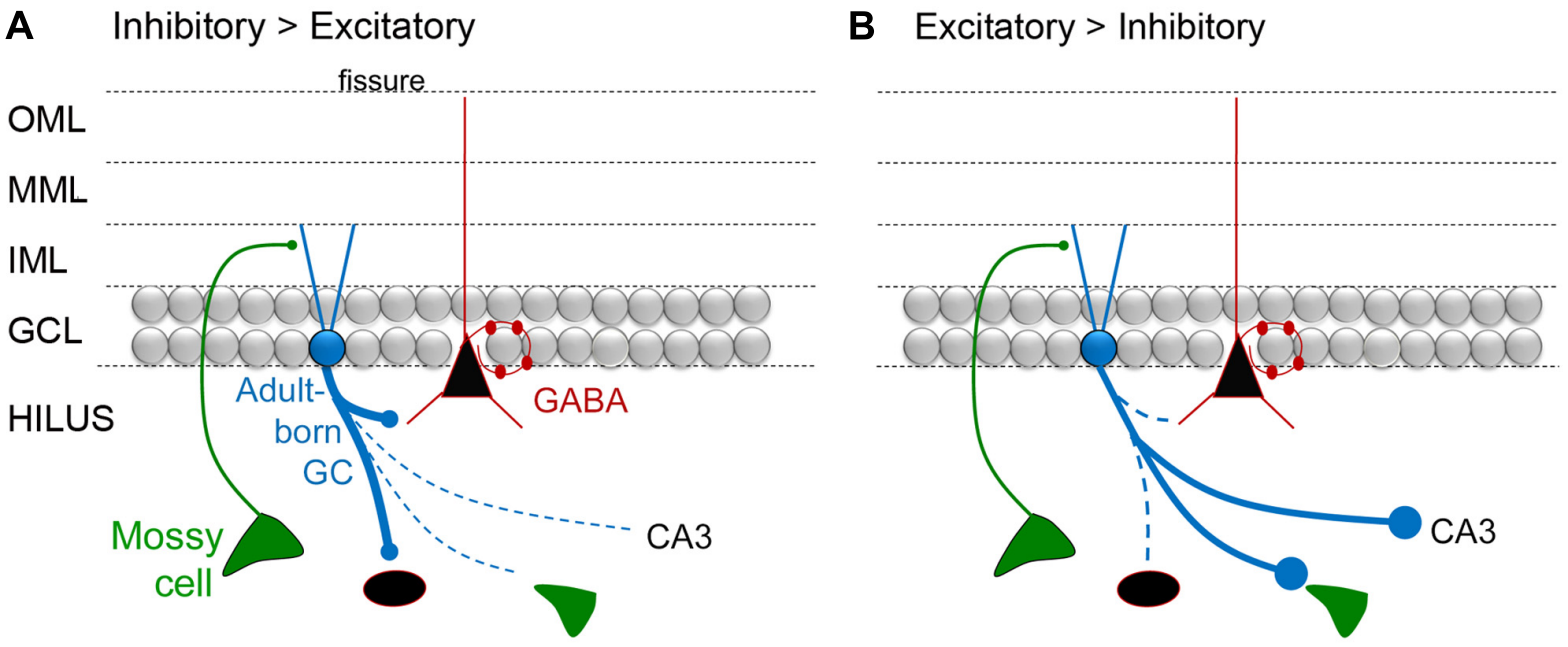
**A diagram of the hypothesis that mossy fiber axons regulate the extent that adult-born GCs are inhibitory or excitatory. (A)** A schematic illustrates the idea that adult-born GCs primarily activate inhibitory neurons when they are young. One of the arguments for this hypothesis is that developing mossy fibers (from GCs born in early life) primarily make small synapses that preferentially contact inhibitory neurons, and then the ‘giant’ boutons that innervate excitatory neurons mature (discussed further in the text). **(B)** Diagrammatic illustration of the idea that adult-born GCs primarily activate glutamatergic cells when they are young. To reflect this idea, the immature GC is shown making large boutons preferentially on excitatory cells such as MCs and CA3 pyramidal cells.

Importantly the maturation of the mossy fiber axon may be a plastic phenomenon, not always the same depending on the age when the GC is born (i.e., neonatal vs. adult), condition (i.e., normal or pathological), and recent experience (i.e., deprivation or enriched environment). That plasticity could change the outcome of the circuit dramatically, either making it strongly inhibitory (if the giant boutons mature very late and are more numerous, contacting more excitatory cells) or much less inhibitory (if the giant boutons mature very rapidly, or they are more numerous, contacting more inhibitory cells; **Figure 5**). Therefore, conditions used in different laboratories to study the effects of adult-born GCs in different animals may lead to a primarily excitatory effect in one experiment and inhibitory effect of adult-born GCs in another experiment (**Figure 5**). This could potentially explain why some investigators find young adult-born neurons produce weak feedback inhibition of mature GCs (Temprana et al., 2015) but others do not (Drew et al., 2013). In support of this explanation, the environment where animals are housed appears to produce very different circuitry of adult-born GCs depending on enrichment, with inhibitory circuitry one of the plastic components of the circuitry (Bergami et al., 2015).

#### The MC → Adult-Born GC Circuit in TLE

The models that simulate TLE typically induce SE to simulate the initial insult. When SE is induced in normal adult rodents, a pattern of cell loss often develops that simulates MTS. MCs are lost, as well as NPY/SOM-expressing GABAergic hilar neurons (Vezzani et al., 1996; Buckmaster and Jongen-Relo, 1999; Sun et al., 2007; Huusko et al., 2015), and there is a rapid increase in the proliferation of new cells in the SGZ (Parent et al., 1997).

**FIGURE 6:**
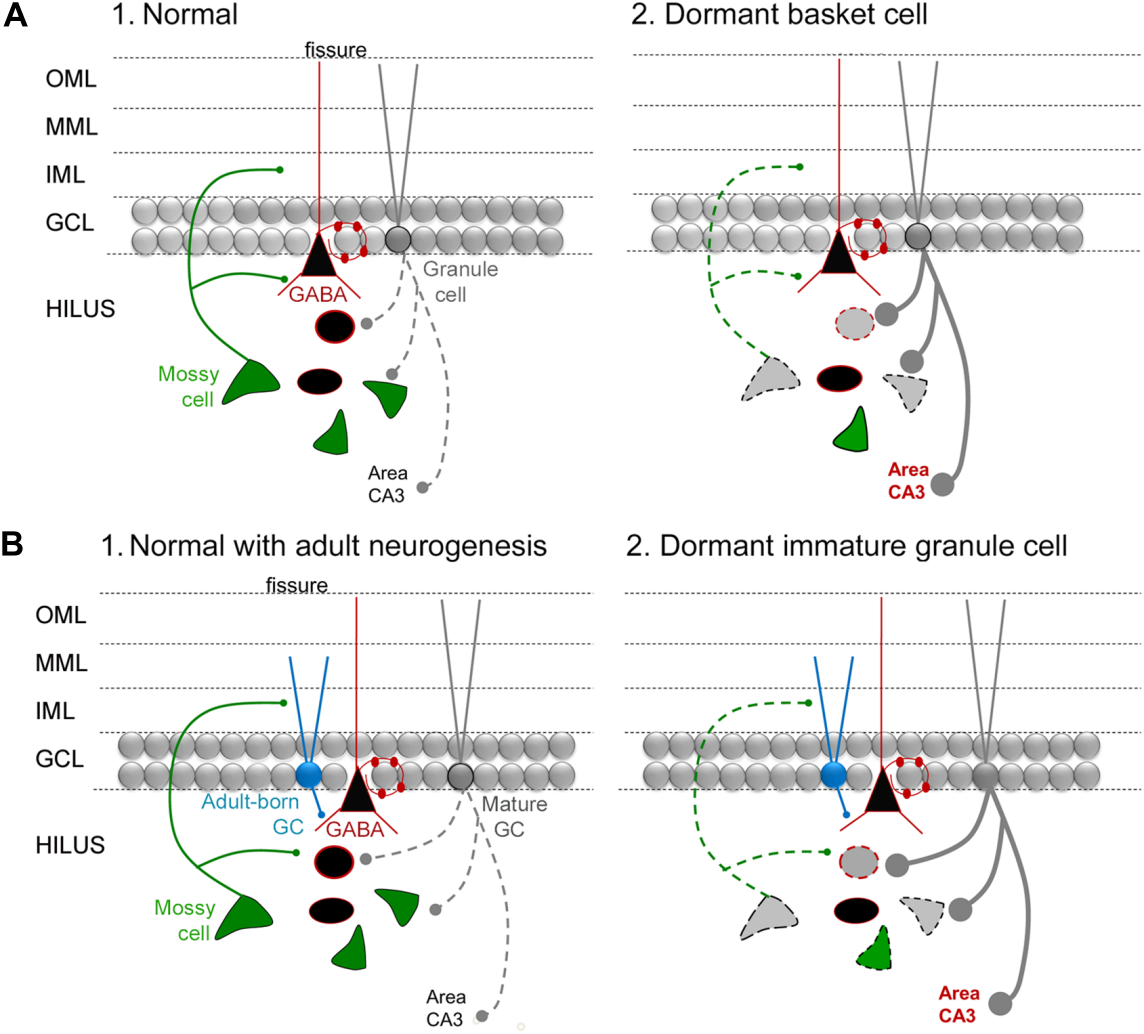
**The dormant immature GC hypothesis. (A)** (1) A diagram of the circuitry of the normal DG is shown. The dotted line reflects the sparse firing of GCs that is considered an important characteristic of the normal DG. (2) The “dormant basket cell hypothesis” (Sloviter, 1991, 1994) proposes that MC loss in TLE (diagrammed by the light gray MC with a dotted line for its axon) leads to deafferentation of basket cells (reflected by the black triangular cell with a red outline) and consequent disinhibition of GCs (reflected by a thick gray line for the axon, with large boutons). Cell loss in TLE includes the MCs and other hilar GABAergic neurons (gray cell bodies with dotted outlines). The red type indicates hyperexcitability in area CA3, which would be an anticipated if there was much greater activity in GCs, and has some experimental support (Scharfman and Schwartzkroin, 1990a,b). **(B)** (1) A diagram of the circuitry of the normal DG, including adult-born GCs that are immature (turquoise) and mature (gray). (2) The dormant immature GC hypothesis proposes that the loss of MCs in TLE leads to dormant adult-born cells during a stage in their development when they are mainly inhibitory because they activate GABAergic neurons preferentially. The outcome is hyperexcitability of GCs and CA3, indicated by the dark gray lines and red type.

For those newborn GCs that develop normally after SE, one would expect that they would help protect the DG from additional seizures or epilepsy in light of the studies of Iyengar et al. (2015) discussed above. MCs may play a role by activating the adult-born GCs, given they provide glutamatergic input to adult-born GCs and do so even when they are young, also discussed above. Importantly, some MCs do survive SE (Buckmaster and Jongen-Relo, 1999; Scharfman et al., 2001; Zhang et al., 2014). However, a large number of MCs are lost. Therefore, adult-born neurons could lose their normal MC afferent input after SE (**Figure 6A**). The result could bethat adult-born GCs become “dormant” or inactive because they are deprived of a major source of excitatory input, when they are young, even if they are otherwise normal. This idea is reminiscent of the idea that MCs normally activate an inhibitory cell type in the DG, basket cells, but if the MCs are reduced in number the basket cells become “dormant” – the “dormant basket cell hypothesis” (**Figure 6**; Sloviter, 1991, 1994; Sloviter et al., 2003). Analogous to the “dormant basket cell hypothesis” one could suggest a “dormant immature-granule cell hypothesis” where MC loss leaves immature GCs with less excitatory afferent input (**Figure 6**). Some support exists for the “dormant immature granule cell hypothesis”: when recordings are made from adult-born GCs after SE they appear to be relatively quiet, rather than active (Jakubs et al., 2006).

The idea that MCs normally activate adult-born GCs – and have an inhibitory effect on the DG network – provides a potential explanation for an observation that has been reported previously. The observation was based on a mouse model of TLE that used intrahippocampal infusion of kainic acid to initiate SE. In this animal model, the ipsilateral hippocampus sustains injury like TLE – the injected hippocampus is shrunken or ‘sclerotic.’ It has been shown that the adult-born GCs are almost completely lost in this damaged hippocampus (Kralic et al., 2005). It has been argued that because of this loss of adult-born GCs, epilepsy is unlikely to be promoted by aberrant adult-born GCs (Kralic et al., 2005). Another interpretation is that the loss of normal adult-born GCs disinhibits the DG, because normally the adult-born GCs participate in a circuit that is inhibitory and reduces seizures. Thus, in the normal DG, adult-born GCs may activate inhibitory neurons and inhibit mature GCs, leading to seizure protection. However, in the epileptic DG after intrahippocampal kainic acid this may not be the case.

## Conclusion

The DG circuits involving MCs are not completely clear, but recent investigations have shed light on potential circuits that have important implications for understanding MC function in health and disease. One study showed that MCs innervate adult-born GCs when the adult-born cells are young. Other studies suggest that adult-born cells are primarily inhibitory when young, although there is debate about this issue at the present time. One implication is that MCs may play a critical role in DG inhibition via the young adult-born GCs. This idea is interesting to consider because it provides a reason why the GCs exhibit sparse firing, an observation that is considered important to DG function. The idea also sheds light on the reasons the DG is a gate for seizures. In TLE, when either MCs, adult-born GCs, or their inhibitory targets are reduced in number, the hypothesis explains why there would be hyperexcitability in the DG and a permissive effect on seizures. Indeed, hyperexcitability has been shown after ablation of MCs, and increased seizure severity has been shown when adult-born GCs are ablated. An initial hypothesis to explain the changes in the DG in TLE was the ‘dormant basket cell hypothesis’ (Sloviter, 1991, 1994; Sloviter et al., 2003) where loss of MCs led to disinhibition because MCs normally innervate basket cells. Here we suggest the ‘dormant immature GC hypothesis’ where loss of MCs leads to disinhibition because MCs normally innervate young adult-born GCs (**Figure 6**).

## Conflict of Interest Statement

The authors declare that the research was conducted in the absence of any commercial or financial relationships that could be construed as a potential conflict of interest.
